# Pregnancy complications and their association with postpartum depression symptoms: a retrospective study

**DOI:** 10.1080/00049530.2023.2247088

**Published:** 2023-08-21

**Authors:** Taliah Swart, Kerrie Shandley, Minh Huynh, Christine M. Brown, David W. Austin, Jahar Bhowmik

**Affiliations:** a School of Health Sciences, Swinburne University of Technology, Hawthorn, VIC, Australia; b School of Psychology, Deakin University, Geelong, VIC, Australia; c Biopsychosocial and e-Health Research and Innovation (BeRI) Hub, Federation University Australia, University Drive, Ballarat, VIC, Australia; d Health Innovation and Transformation Centre, Federation University Australia, University Drive, Ballarat, VIC, Australia; e School of Allied Health, Human Services and Sport, La Trobe University, Bundoora, VIC, Australia

**Keywords:** Postpartum depression, pregnancy complications, cytomegalovirus, foetal distress, emergency caesarean, placenta previa

## Abstract

**Objective:**

Postpartum depression affects around 17% of the women worldwide and has considerable implications for maternal and child health. While some risk factors have been identified, the association between pregnancy and delivery complications and postpartum depression is less well understood. This study aims to determine whether specific pregnancy complications are associated with risk of postpartum depression symptoms (PPDS).

**Method:**

This study analysed a subset of variables collected as part of a larger study exploring pregnancy circumstances and maternal-foetal health outcomes. Mothers residing in Australia provided information on their biological children aged 3–13 years. Pregnancy complications were analysed using bivariate analyses and binary logistic regression.

**Results:**

Mothers (*N* = 1,926) reported on *N* = 3,210 pregnancies (mean number of pregnancies = 1.27, SD = 0.97). At the time of childbirth, mothers were on average 30.1 years old (SD = 5.14). Experiencing a pregnancy complication increased the risk of PPDS (X^2^ = 16.45, df = 1, *p* < 0.001) However, logistic regression analyses indicated an increased risk of PPDS was associated with the specific pregnancy complications of cytomegalovirus (AOR = 7.06, 95% CI[1.51,32.98]), emergency caesarean (AOR = 1.67, 95% CI[1.31,2.12]), foetal distress before birth (AOR = 1.49, 95% CI[1.16,1.91]), induced labour (AOR = 1.55, 95% CI[1.25,1.91]) and placenta previa (AOR = 2.60, 95% CI[1.44,4.71]).

**Conclusion:**

Specific pregnancy complications were associated with PPDS, suggesting that some complications may pose a greater risk for PPDS than others. This study contributes to the growing understanding of peripartum risk factors for postpartum depression, and suggests that early clinical identification of at-risk mothers and early prophylactic and supportive care may be warranted to reduce that risk.

## Key Points

**What is already known about this topic**:

1. Postpartum depression is a prevalent mental health condition affecting almost 1 in 5 mothers worldwide.
2. A complicated pregnancy and delivery experience is associated with an increased risk of adverse psychosocial outcomes, including postpartum depression.
3. Targeted interventions in the peripartum period for mothers identified to be at higher risk of postpartum depression may be effective at mitigating the impact.

**What this topic adds**:

1. Analysis of distinct and specific pregnancy complications against postpartum depression symptoms revealed that certain complications had a more significant impact than others in an Australia-based maternal cohort.
2. Emergency caesarean delivery, cytomegalovirus, foetal distress, induced labour, and placenta previa were significant predictors of postpartum depression symptom development.
3. Gestational diabetes, bleeding during pregnancy, low amniotic-fluid index, pre-eclampsia, and candida infections were not associated with postpartum depression symptoms in this study.

## Introduction

Depression in the postpartum period is a pervasive mental health concern, with a global prevalence of 17% (Wang et al., 2021). The DSM-5-TR (American Psychiatric Association [APA], 2022) does not recognise postpartum depression as a unique diagnostic classification, but rather as Major Depressive Disorder with a postpartum onset. It is specified that the onset of a depressive episode must occur within four weeks of childbirth, however, it has been argued that the timeframe is too restrictive, with 6- to 12-months more commonly accepted internationally (American College of Obstetricians and Gynecologists, 2015; Austin, 2014). Diagnostic criteria differentiating this condition from clinical depression include sleep disturbance, weight change, unreasonable feelings of guilt and self-reproach, reduced confidence and loss of energy separate from associated depression (APA, 2022). Postpartum depression has been linked to compromised functioning and interpersonal relations for mothers, disrupted maternal-foetal bonding and long-term adverse outcomes for the child (Brake et al., 2020). Sociodemographic and postpartum variables have been extensively researched for their impact on maternal postpartum depression risk in comprehensive reviews (Gastaldon et al., 2022; Ghaedrahmati et al., 2017), but research on pregnancy-related and peripartum risk factors needs elucidation.

Pregnancy complications significantly impact the physical and mental health of the mother and child, yet little research exists on the direct risk of pregnancy complications on postpartum depression. A large-scale systematic review by Biaggi et al. (2016) and an umbrella review by Hutchens and Kearney (2020) found the risk of depression in the postpartum period to be increased among mothers who had experienced pregnancy and/or obstetric complications; however, these studies investigated pregnancy complications in aggregate (whether or not pregnancy and/or obstetric complications were present) rather than isolating distinct complications. As there is some nuance to how pregnancy complications affect maternal health outcomes, specific complications may confer greater risk. A prospective longitudinal study conducted in the Netherlands (Blom et al., 2010) found suspicion of foetal distress before birth to significantly increase postpartum depression risk, and negative delivery experiences (e.g., induced labour and emergency caesarean) were similarly associated with postpartum depression in an umbrella review (Gastaldon et al., 2022), a Dutch prospective study (Van der Zee Van den Berg et al., 2021) and a study examining data extracted from the Korean National Health Insurance Service-National Sample Cohort (Nam et al., 2017). Additionally, systematic reviews have found gestational diabetes (Azami et al., 2019) and pre-eclampsia (Caropreso et al., 2020) to be associated with postpartum depression occurrence. A review by Disha et al. (2022) found that adverse pregnancy events are also associated with maternal-foetal infection, which may implicate conditions such as viral candidiasis and cytomegalovirus (CMV), particularly in recognised high-risk pregnancies such as in oligohydramnios (low amniotic fluid) (Taneja et al., 2017).

The implementation of interventions, such as increased social and parental support, therapeutic resources and programs and targeted psychological interventions, have demonstrated improved outcomes for mothers and families affected by postpartum depression (Werner et al., 2014; Van der Waerden et al., 2019). Improved identification of mothers at-risk of postpartum depression may create new preventive intervention pathways in the peripartum period.

This study aims to investigate whether specific pregnancy complications contribute to an increased risk of postpartum depression symptoms (PPDS) in mothers. Gestational diabetes, candidiasis, oligohydramnios, bleeding during pregnancy, CMV, pre-eclampsia, foetal distress before birth, induced labour and emergency caesarean delivery were investigated for their potential association with maternal PPDS.

## Methods

### Participants and procedures

This study analysed a subset of data collected as part of a larger retrospective investigation into pregnancy, birth circumstances, and maternal-foetal health outcomes. One paper has been published based upon a different subset of variables from this survey, investigating risk factors for autism spectrum disorder (Whitely et al., 2021). Following ethical approval from Swinburne University, an online survey was advertised in an Australian parenting magazine, on parenting support forums, and at a homebirth conference in Victoria, Australia. Biological mothers who had at least one child aged 3–13 years were invited to participate. The age range reflected requirements in the original study to 1) encapsulate the typical diagnostic age range of autism spectrum disorder and 2) to minimise the effect of memory bias among respondents. While advertising was largely directed at an Australian audience, it did not preclude the participation of mothers residing internationally.

### Measures

The survey comprised four sections asking after the demographic and health information of the biological parents, pregnancy histories of the biological mothers, birth and early feeding history of each participating child and health outcomes of each participating mother and child. The survey asked mothers to identify whether they had experienced various complications during or following birth from a list by ticking a checkbox for each reported pregnancy/birth. Additionally, an open text box was provided for the mother to specify any other complications experienced that were not included in the list. For the outcome variable of postpartum depression, it was noted that some women had self-diagnosed experiencing depression following the birth of their child. As it was not possible to separate women who self-diagnosed postpartum depression versus those who were formally diagnosed, we refer to the outcome variable as postpartum depression symptoms (PPDS), which is defined as the presence/absence of formal or self-diagnosed postpartum depression.

PPDS was characterised as a binary variable (0 = no, 1 = yes), as were the following predictors: bleeding during pregnancy, CMV, emergency caesarean, induced labour, gestational diabetes, oligohydramnios, placenta previa, pre-eclampsia, multiple birth delivery and candida infections. An overall “pregnancy complications” binary variable was created, where a “yes” was defined as the presence of one or more pregnancy or obstetric complications endorsed from the list provided in the survey (bleeding during pregnancy, CMV, gestational diabetes, oligohydramnios, placenta previa, preeclampsia and candida infection) and/or “other” complication/s entered via text box (e.g., anaemia, obstetric cholestasis, hypertension). Conversely, a “no” response was defined by the absence of any pregnancy complications endorsed or entered in the text box by the mother.

An age split was introduced to maternal age at birth (advanced maternal age ≥35 years), with advanced maternal age quantified as a binary variable (0 = no, 1 = yes). The age split is reflective of Australian clinical standards for advanced maternal age classification (Australian Institute of Health and Welfare, 2021). A binary multiple births variable (0 = no, 1 = yes) was also created by combining all cases of twins and triplets. Foetal distress before birth had three responses (0 = no, 1 = yes, 2 = unknown), as did education level (0 = year 12 or lower, 1 = trade/diploma, 2 = University) and current employment of the mother (0 = unemployed/unpaid work [including voluntary and at-home duties], 1 = employed, 2 = student).

### Statistical analysis

Multicollinearity among the predictors was examined through the variance inflation factor (VIF). The VIF values for this dataset was less than 3, indicating no strong correlation was present between the predictors. Chi-square statistics were performed to identify the associations between specific pregnancy complications and PPDS, and Cramér’s *V* to quantify the size of the effect, where values greater than 0.5 indicated a moderate-to-large effect and values below 0.1 were considered weak. A multivariate binary logistic regression was conducted to determine the effect of pregnancy complications against PPDS occurrence, with the statistical significance level set to 0.05. Model fit was evaluated using the Hosmer-Lemeshow test, pseudo R^2^ and model classification statistics. The data were analysed using IBM SPSS (v.28).

## Results

### Mother and pregnancy characteristics

In total, 2,473 biological mothers responded to the survey, providing data on 4,306 pregnancies. Pregnancies where the child was aged outside the 3–13 years of age criteria at the time of survey completion (*n* = 223) were excluded from analysis in accordance with the inclusion criteria set by the original study. For analysis, only complete sets of data were retained with 820 incomplete cases excluded, for a completion rate just over 80%. The remaining pregnancies (*N* = 3,263) comprised 3,105 singletons, 51 sets of twins and one set of triplets. For analysis, each set of multiple births was treated as a single pregnancy (rather than two or three separate pregnancies for twins and triplets respectively). This resulted in a final sample of 1,926 mothers reporting on 3,210 pregnancies.

Most mothers (*n* = 2511, 78.2%) resided in Australia, followed by the United States (*n* = 338, 110.5%), Canada (*n* = 148, 4.6%) and the United Kingdom (*n* = 119, 4.6%). Mothers reported on an average of 1.7 pregnancies (SD = 0.82, range = 1–5). At the time of childbirth, mothers had an average age of 30.1 years (SD = 5.14, age range = 15–46). The children had an average age of 6.3 years (SD = 2.84, age range = 3–13) at the time of the survey. Of the *N* = 3,210 pregnancies included in the study, PPDS was reported by mothers in association with 16.5% (*n* = 531) of the pregnancies. Table 1 displays the characteristics of the mothers and pregnancies for the total sample (*N* = 3,210) and PPDS subgroup (*n* = 531).

**Table 1.** Participant characteristics for the total sample (*N* = 3,210) and PPDS subgroup (*n* = 531) and results of the bivariate analyses between sociodemographic factors and pregnancy complications against mothers PPDS.

		Postpartum depression symptoms, n (%)			
	Total sample (n (%)	Yes	No	*p*-value	Cramér’s V
Sociodemographic factors					
Maternal age at childbirth				0.022	0.041
≥35 years of age	656 (20.4)	89 (16.8)	442 (83.2)		
Education level				0.069	0.041
Year 12 and below	464 (14.5)	91 (17.1)	440 (82.9)		
Trade, Diploma or TAFE	815 (25.4)	142 (26.7)	389 (73.3)		
University level	1931 (60.2)	298 (56.1)	233 (43.9)		
Mother employment status				0.048	0.048
Employed	2050 (63.9)	316 (59.5)	215 (40.5)		
Unemployed/Unpaid	1085 (33.8)	204 (38.4)	327 (61.6)		
Student	75 (2.3)	11 (2.1)	520 (97.9)		
Multiple births	52 (1.6)	16 (3.0)	515 (97.0)	0.013	0.049
Predictors					
Pregnancy complications	1162 (36.2)	233 (43.9)	298 (56.1)	<0.001	0.071
Bleeding during pregnancy	378 (11.8)	77 (14.5)	454 (85.5)	0.039	0.038
CMV	7 (0.2)	4 (0.8)	527 (99.2)	0.017	0.051
Emergency caesarean section	519 (16.2)	135 (25.4)	396 (74.6)	<0.001	0.112
Foetal distress before birth	508 (15.8)	131 (24.7)	400 (75.3)	<0.001	0.108
Gestational diabetes	120 (3.7)	25 (4.7)	506 (95.3)	0.210	0.023
Induced labour	817 (25.5)	187 (35.2)	344 (64.8)	<0.001	0.100
Oligohydramnios	51 (1.6)	14 (2.6)	517 (97.4)	0.054	0.037
Placenta previa	56 (1.7)	20 (3.8)	511 (96.2)	<0.001	0.069
Preeclampsia	129 (4.0)	28 (5.3)	503 (94.7)	0.115	0.028
Candidiasis	411 (12.8)	80 (15.1)	451 (84.9)	0.088	0.030

CMV = Cytomegalovirus.

^a^Frequency and percentage of “yes” responses for the total sample (*N* = 3,210).

^b^Frequency and percentage of “yes” and “no” responses to sociodemographic and pregnancy-and-obstetric complications in the subgroup of participants where PPDS was reported as present (*n* = 531).

^c^Pregnancies where one or more complication was present during a single pregnancy.

### Binary logistic regression

The association between sociodemographic variables and pregnancy complications with PPDS was explored using bivariate analyses (chi-square tests and Cramér’s *V*). The results of these analyses are presented in Table 1, and show that advanced maternal age, multiple-birth delivery, bleeding during pregnancy, CMV, emergency caesarean, foetal distress before birth, induced labour, oligohydramnios and placenta previa were significant, however, all Cramér’s *V* effect sizes were below 0.1 except induced labour (*V* = 0.100), emergency caesarean (*V* = 0.112) and foetal distress before birth (*V* = 0.108) indicating a small effect on PPDS. The results also demonstrated that there was a significant association between pregnancy complications as a singular variable and PPDS (χ^2^ = 16.45, *df* = 1, *p* < 0.001, *V* = 0.071). A subsequent binary logistic regression was conducted to investigate the effect of individual pregnancy complications on PPDS, with the results displayed in Table 2.

**Table 2.** Associations between specific pregnancy complications and postpartum depression symptoms: results from the multivariate binary logistic regression model.

	Slope (Beta coefficient)	AOR (95% CI)	Wald Statistic	*p*-value
Sociodemographic factors				
Advanced maternal age (≥35 years of age)	0.33	0.72 (0.56,0.93)	6.40	0.011
Education level^a^				
Trade, Diploma, TAFE	0.15	1.16 (0.88,1.52)	1.15	0.283
University Degree	0.05	1.05 (0.84,1.32)	0.21	0.651
Employment status^b^				
Employed	0.23	0.80 (0.65,0.97)	4.98	0.026
Unemployed/Unpaid	0.22	0.80 (0.41,1.57)	0.42	0.517
Multiple births	0.83	2.28 (1.23,4.24)	6.81	0.009
Predictors				
Pregnancy complications^c^	0.14	1.15 (0.84,1.56)	0.74	0.390
Bleeding during pregnancy	0.04	1.04 (0.74, 1.45)	0.04	0.838
CMV	1.95	7.06 (1.51,32.98)	6.18	0.013
Emergency caesarean section	0.51	1.67 (1.31, 2.12)	17.46	<0.001
Foetal distress before birth	0.40	1.49 (1.16, 1.91)	9.86	0.0002
Gestational diabetes	0.01	0.99 (0.60, 1.62)	0.00	0.964
Induced Labour	0.44	1.55 (1.25, 1.91)	16.30	<0.001
Oligohydramnios	0.34	1.41 (0.72,2.76)	0.99	0.321
Placenta previa	0.96	2.60 (1.44,4.71)	10.05	0.002
Preeclampsia	0.11	0.90 (0.55, 1.45)	0.12	0.657
Candidiasis	0.153	1.17 (0.83, 1.64)	0.77	0.381
Constant	1.87	0.154	279.9	<0.001

CMV = Cytomegalovirus; AOR = Adjusted Odds Ratio; CI = Confidence Interval; Wald Statistic = Wald chi-square test.

^a^Year 12 or below is treated as the reference category.

^b^Student is treated as the reference category.

^c^Pregnancies where one or more complication was present during a single pregnancy.

Hosmer and Lemeshow goodness-of-fit results indicated that the regression model was a good fit for the data, χ^2^ (7) = 6.243, *p* = 0.512. The pseudo R^2^ values were relatively low (Cox and Snell = 0.035, Nagelkerke = 0.059), however, the model correctly classified 83.4% of cases. A significant decrease in PPDS risk was identified for mothers who were employed at the time of survey completion (AOR = 0.80, 95% CI[0.65,0.97]), as well as for advanced maternal age (AOR = 0.72, 95% CI[0.56,0.93]). PPDS was significantly more likely to be associated with a multiple birth pregnancy than singleton pregnancy (AOR = 2.28, 95% CI[1.23,4.24]).

The predictors that were significantly associated with PPDS risk in the regression model included CMV (AOR = 7.06, 95% CI[1.51,32.98]), emergency caesarean section (AOR = 1.67, 95% CI[1.31,2.12]), foetal distress before birth (AOR = 1.49, 95% CI[1.16,1.91]), induced labour (AOR = 1.55, 95% CI[1.25,1.91]) and placenta previa (AOR = 2.60, 95% CI[1.44,4.71]). Gestational diabetes (AOR = 0.99, 95% CL[0.60,1.62]), bleeding during pregnancy (AOR = 1.04, 95% CI[0.74,1.45]), oligohydramnios (AOR = 1.41, 95% CI[0.72,2.76]), pre-eclampsia (AOR = 0.90, 95% CI[0.55,1.45]), and candida infections (AOR = 1.17, 95% CI[0.83,1.64]) were not significantly associated with PPDS in this study, neither was pregnancy complications as a singular variable (AOR = 1.15, 95% CI[0.84, 1.56]).

## Discussion

This study explored the association between specific pregnancy complications and PPDS occurrence. Several pregnancy complications significantly predicted PPDS, namely CMV, emergency caesarean, foetal distress before birth, induced labour and placenta previa. Maternal employment and advanced maternal age significantly reduced PPDS risk whereas a multiple birth delivery was associated with over twice the risk of PPDS.

While pregnancy complications as a single variable was associated with PPDS, in line with prior reviews (Biaggi et al., 2016; Hutchens & Kearney, 2020), it was not a significant predictor in the multivariate model. This may indicate that some specific pregnancy complications pose a greater risk than others. Notably, mothers who had CMV presented with over seven-times the risk of PPDS compared to mothers who did not; to our knowledge, this direct relationship has not previously been explored. Placenta previa significantly predicted the occurrence of PPDS, which accords with a recent cross-sectional study in Taiwan (Lin et al., 2022), but this association otherwise does not appear to have been fully investigated in the published literature.

Consistent with the literature (Blom et al., 2010; Gastaldon et al., 2022; Nam et al., 2017), foetal distress and induced labour increased PPDS risk. An association between altered birth plan (such as induced or emergency delivery) and negative maternal psychosocial outcomes, including postpartum depression, was discussed by Benton et al. (2019); this study may provide further support for this relationship. Emergency caesarean likewise increased risk of PPDS occurrence in this cohort, which is consistent with findings from a larger review study (Gastaldon et al., 2022), a retrospective study (Tonei, 2019) and a prospective investigation into the effect of unplanned caesarean on mother-and-infant bonding and consequent adverse postpartum depressive outcomes (Doblin et al., 2023). Contrary to expectations, gestational diabetes was not associated with a significantly increased risk of PPDS in this study, nor were candida infections. However, the incidence of these predictors was lower in this sample compared with Australian population rates (Australian Institute of Health and Welfare, 2019), consequently there may have been insufficient power to detect an association.

These results need to be viewed in consideration of the study limitations. The study was based on a retrospective, self-report survey on pregnancy and obstetric histories and consequently, recall bias may have been a factor. Also, mothers were asked whether they experienced postpartum depression as a complication following the birth of their child/children, rather than whether they were formally diagnosed with postpartum depression. Therefore, it was not possible to separate respondents who were formally diagnosed with postpartum depression from those who self-diagnosed. Underdiagnosis of postpartum depression due to a multitude of factors including comorbid hormonal fluctuations, disrupted sleep and stigma-associated under-reporting is a notable, ongoing concern in maternal healthcare (Thomson & Sharma, 2017). Therefore, the aforementioned limitations may potentially be more inclusive of mothers affected by depression symptoms following childbirth. Additionally, maternal access to pregnancy and birthing health records may have been limited, and as the survey was anonymous, it was not possible to cross-reference the self-reported data with hospital records. Finally, mothers were not asked whether they had previously received a postpartum depression diagnosis for a pregnancy that fell outside the inclusion criteria of the original study. Consequently, it was not possible to control for a prior diagnosis of postpartum depression in the analysis.

Future studies would benefit from exploring additional, potentially confounding and/or mediating factors such as indicators of lower socioeconomic status, infant health conditions, antepartum maternal mental health, preterm labour, common infections and the effect of severity and co-occurrence of obstetric complications. Nevertheless, this study contributes to the knowledge base of peripartum risk factors for postpartum depression and suggests that early identification of at-risk mothers and efforts to reduce the likelihood of postpartum depression may be warranted.

## Data Availability

The de-identified data that support the findings of this study are available from the corresponding author (JB) upon reasonable request and following Swinburne University HREC ethical approval to do so.

## References

[cit0001] The American College of Obstetricians and Gynecologists Committee Opinion. (2015). Screening for perinatal depression. *Journal of Obstetrics and Gynecology*, 125(630), 1268–7. 10.1097/01.AOG.0000465192.34779.dc 25932866

[cit0002] American Psychiatric Association. (2022). *Diagnostic and statistical manual of mental disorders* *text rev* (DSM-5-TR) (5th ed.). American Psyschiatric Publishing. 10.1176/appi.books.9780890425787

[cit0003] Austin, M. P., & Marce Society Position Statement Advisory Committee. (2014). Marce International Society position statement on psychosocial assessment and depression screening in perinatal women. *Best Practice and Research Clinical Obstetrics & Gynaecology*, 28(1), 179–187. 10.1016/j.bpobgyn.2013.08.016 24138943

[cit0004] Australian Institute of Health and Welfare. (2019). Incidence of gestational diabetes in Australia. https://www.aihw.gov.au/reports/diabetes/incidence-of-gestational-diabetes-in-australia

[cit0005] Australian Institute of Health and Welfare. (2021). Older mothers in Australia. https://www.aihw.gov.au/reports/mothers-babies/older-mothers-in-australia-2019/summary

[cit0006] Azami, M., Badfar, G., Soleymani, A., & Rahmati, S. (2019). The association between gestational diabetes and postpartum depression: A systematic review and meta-analysis. *Diabetes Research and Clinical Practice*, 149, 147–155. 10.1016/j.diabres.2019.01.034 30735772

[cit0007] Benton, M., Salter, A., Tape, N., Wilkinson, C., & Turnbull, D. (2019). Women’s psychosocial outcomes following an emergency caesarean: A systematic literature review. *BMC Pregnancy and Childbirth*, 19(535). 10.1186/s12884-019-2687-7 PMC6937939 31888530

[cit0008] Biaggi, A., Conroy, S., Pawlby, S., & Pariante, C. M. (2016). Identifying the women at risk of antenatal anxiety and depression: A systematic review. *Journal of Affective Disorders*, 191, 62–77. 10.1016/j.jad.2015.11.014 26650969 PMC4879174

[cit0009] Blom, E. A., Jansen, P. W., Verhulst, F. C., Hofman, A., Raat, H., Jaddoe, V. W. V., Coolman, M., Steegers, E., & Tiemeier, H. (2010). Peripartum complications increase the risk of postpartum depression: The generation R study. *Journal of Obstetrics and Gynaecology*, 117(11), 1390–1398. 10.1111/j.1471-0528.2010.02660.x 20682022

[cit0010] Brake, E., Berle, D., Reilly, N. M., & Austin, M. (2020). The relationship between emotion dysregulation and postpartum attachment in women admitted to a mother-baby unit. *Australian Journal of Psychology*, 72(3), 282–292. 10.1111/ajpy.12289

[cit0011] Caropreso, L., Cardoso, T. A., Eltayebani, M., & Frey, B. N. (2020). Preeclampsia is a risk factor for postpartum depression and psychosist: A systematic review and meta-analysis. *Archives of Women’s Mental Health*, 24, 493–505. 10.1007/s00737-019-01010-1 31802249

[cit0012] Disha, T., Haque, F., & Baig, A. A. (2022). Prevalence and risk factors of vulvaginal candidosis during pregnancy: A review. *Infectious Diseases in Obstetrics and Gynecology*, 1–14. 10.1155/2022/6195712 PMC9329029 35910510

[cit0013] Doblin, S., Seefeld, L., Weise, V., Kopp, M., Knappe, S., Asselmann, E., Martini, J., & Garthus-Niegel, S. (2023). The impact of mode of delivery on parent-infant-bonding and the mediating role of birth experience: A comparison of mothers and fathers within the longitudinal cohort study DREAM. *BMC Pregnancy and Childbirth*, 23(285). 10.1186/s12884-023-05611-8 PMC10127505 37098555

[cit0014] Gastaldon, C., Solmi, M., Correll, C. U., Barbui, C., & Schoretsanitis, G. (2022). Risk factors of postpartum depression and depressive symptoms: Umbrella review of current evidence from systematic reviews and meta-analyses of observational studies. *British Journal of Psychiatry*, 1(4), 591–602. 10.1192/bjp.2021.222 35081993

[cit0015] Ghaedrahmati, M., Kazemi, A., Kheirabadi, G., Ebrahimi, A., & Bahrami, M. (2017). Postpartum depression risk factors: A narrative review. *Journal of Education and Health Promotion*, 6(60). 10.4103/jehp.jehp_9_16 PMC5561681 28852652

[cit0016] Hutchens, B. F., & Kearney, J. (2020). Risk factors for postpartum depression: An umbrella review. *Journal of midwifery & women's health*, 65(1), 96–108. 10.1111/jmwh.13067 31970924

[cit0017] Lin, Y., Chen, C., Sun, F., & Chen, C. (2022). Risk and protective factors related to immediate postpartum depression in a baby-friendly hospital in Taiwan. *Taiwanese Journal of Obstetrics & Gynecology*, 61(6), 977–983. 10.1016/j.tjog.2022.08.004 36428001

[cit0018] Nam, J. W., Choi, Y., Kim, J., Cho, K. H., & Park, E. C. (2017). The synergistic effect of breastfeeding discontinuation and caesarean section delivery on postpartum depression: A nationwide population-based cohort study in Korea. *Journal of Affective Disorders*, 218, 53–58. 10.1016/j.jad.2017.04.048 28458116

[cit0019] Taneja, A., Arora, K., Chopra, I., & Naik, S. S. (2017). Pregnancy outcomes in isolated oligohydramnios during second trimester: A case series. *Journal of Clinical & Diagnostic Research*, 11(8). 10.7860/2FJCDR/2F2017/2F27722.10502 PMC5620861 28969220

[cit0020] Thomson, M., & Sharma, V. (2017). Therapeutics of postpartum depression. *Expert Review of Neurotherapeutics*, 17(5), 495–507. 10.1080/14737175.2017.1265888 27892736

[cit0021] Tonei, V. (2019). Mother’s mental health after childbirth: Does the delivery method matter? *Journal of Health Economics*, 63, 182–196. 10.1016/j.jhealeco.2018.11.006 30594609

[cit0022] Van der Waerden, J., Nakamura, A., Pryor, L., Charles, M., El-Khoury, F., Dargent-Molina, P., & EDEN Mother–Child Cohort Study Group. (2019). Domain-specific physical activity and sedentary behaviour during pregnancy and postpartum depression risk in the French EDEN and ELFE cohorts. *Preventive Medicine*, 121, 33–39. 10.1016/j.ypmed.2019.02.012 30763624

[cit0023] Van der Zee Van den Berg, A. I., Boere-Boonekamp, M. M., Groothuis-Outshoorn, C. G. M., & Reijneveld, S. A. (2021). Postpartum depression and anxiety: A community-based study on risk factors before, during and after pregnancy. *Journal of Affective Disorders*, 286, 158–165. 10.1016/j.jad.2021.02.062 33725615

[cit0024] Wang, Z., Liu, J., Shuai, H., Cai, Z., Fu, X., Liu, Y., Xiao, X., Zhang, W., Krabbendam, E., Liu, S., Liu, Z., Li, Z., & Yang, B. X. (2021). Mapping global prevalence of depression among postpartum women. *Translational Psychiatry*, 11(1). 10.1038/s41398-021-01663-6 PMC8528847 34671011

[cit0025] Werner, E., Miller, M., Osborne, L. M., Kuzava, S., & Monk, C. (2014). Preventing postpartum depression: Review and recommendations. *Archives of Womens Mental Health*, 18(1), 41–60. 10.1007/s00737-014-0475-y PMC4308451 25422150

[cit0026] Whitely, A., Shandley, K., Huynh, M., Brown, C. M., Austin, D. W., & Bhowmik, J. (2021). Brief report: pregnancy, birth and infant feeding practices: A survey-based investigation into risk factors for autism spectrum disorder. *Journal of Autism and Developmental Disorders*, 52(11), 5072–5078. 10.1007/s10803-021-05348-3 34766207

